# The Long‐Term Journey of a Tuberculosis Patient With Triple Organ Involvement and Rheumatological Disease: A Case Study and Literature Review

**DOI:** 10.1002/ccr3.9614

**Published:** 2024-12-12

**Authors:** Homina Saffar, Pooria Sobhanian, Shahriar Alian, David Darvishnia, Mansoureh Baradaran

**Affiliations:** ^1^ Student Research Committee, Faculty of Medicine Mazandaran University of Medical Sciences Sari Mazandaran Iran; ^2^ Department of Infectious Diseases, School of Medicine, Antimicrobial Resistance Research Center, Communicable Diseases Institute, Ghaem Shahr Razi Hospital Mazandaran University of Medical Sciences Sari Mazandaran Iran; ^3^ Faculty of Medicine Mazandaran University of Medical Sciences Sari Mazandaran Iran; ^4^ Department of Radiology, Imam Ali Hospital North Khorasan University of Medical Science Bojnurd North Khorasan Iran

**Keywords:** Behçet's disease, brain abscess, extrapulmonary TB, *Mycobacterium tuberculosis*, pulmonary TB

## Abstract

Tuberculosis (TB) is an infectious disease caused by *Mycobacterium tuberculosis* bacteria, which is more prevalent among immunocompromised individuals. According to the distribution of affected organs, this infection can be categorized as either pulmonary or extrapulmonary TB. Immunodeficiency states resulting from rheumatological disorders and the use of immunosuppressive medications, such as in Behçet's disease (BD), are potential predisposing factors for TB, particularly in cases involving multiple organs. These situations can introduce challenges in both the diagnosis and treatment of patients. We describe a 43‐year‐old man with a history of BD who presented with symptoms of weight loss, abdominal pain, and shortness of breath. His chest X‐ray revealed cavities and calcifications, while an abdominal X‐ray demonstrated signs of intestinal obstruction and adhesions. Subsequent TB diagnosis led to a 6‐month course of a TB treatment regimen. Despite treatment initiation, the patient developed a brain abscess 1 year later, necessitating surgical intervention. Following the procedure, he received another 1‐year course of a TB treatment regimen and experienced full recovery without any complications during a 2‐year follow‐up period. Notably, the patient recently received a Sinopharm COVID‐19 vaccine and subsequently developed seizures that are currently being managed with anticonvulsant therapy. This case report emphasizes the significance of including pulmonary TB in complex medical cases, especially in individuals with autoimmune diseases. Early diagnosis and treatment are crucial for improving outcomes and reducing the risk of complications. Furthermore, it highlights the possible correlation between TB and BD, along with the potential adverse reactions to COVID‐19 vaccines in this population, which necessitate special consideration by healthcare professionals.

**Summary**

- Early recognition and treatment of tuberculosis, particularly in patients with autoimmune diseases like Behcet's disease, are essential for optimal outcomes and to prevent complications.
- Clinicians should maintain a high index of suspicion for TB in complex cases to ensure timely intervention and management.

## 1 Introduction

Tuberculosis (TB) is an infectious disease caused by *Mycobacterium tuberculosis* bacteria, which has afflicted humanity since ancient times and resulted in numerous fatalities. In 2021, a total of 10.6 million cases of active TB and 1.6 million deaths were reported globally [1]. TB is more common among immunocompromised individuals, with the incidence of active TB in patients with rheumatic diseases estimated at 882 per 100,000 and in Behçet's disease (BD) at approximately 30% [2, 3]. TB can affect various organs, including the lungs, pleura, lymph nodes, skin, gastrointestinal tract (GI), joints and bones, and even the central nervous system (CNS). Patient classification is based on the pattern of organ involvement, with lung‐involvement cases termed pulmonary TB, while those affecting other organs are categorized as extrapulmonary TB. Hematogenous dissemination of *M. tuberculosis* bacteria and multi‐organ involvement can lead to a particularly severe and potentially fatal form of disease known as miliary TB [4, 5].

Immune deficiencies and consumption of immunosuppressive drugs can make patients susceptible to infections, including TB, which is a recognized risk factor for the reactivation of latent TB infection (LTBI). Additionally, BD has been associated with TB development through similar mechanisms. BD is characterized by ophthalmic, cutaneous, genital, and aphthous manifestations, and can also affect the CNS, GI, and musculoskeletal systems [3].

TB is diagnosed using a variety of methods, including culture, biopsy, immunological tests, and analysis of body fluids. However, diagnosing the disease can be challenging because of its non‐specific manifestations, as well as bacterial culture difficulties. Sputum or tissue culture, as well as the tuberculin skin test, are commonly used to diagnose TB. Other diagnostic techniques such as chest X‐rays can also aid in diagnosis, although their accuracy is relatively low, particularly in cases of extrapulmonary TB or in immunocompromised patients [4, 6].

TB is treated with a multi‐drug regimen consisting of four antibiotics: pyrazinamide, isoniazid, rifampin, and ethambutol. The treatment aims to improve patient outcomes, prevent transmission of the disease, and reduce the emergence of treatment‐resistant TB [4, 6]. In some cases, surgical removal of infected and necrotic tissue is required, as well as additional complication management [4, 5, 6].

In this case report, we present a 43‐year‐old patient with underlying BD diagnosed with pulmonary and extrapulmonary TB. Given the atypical clinical presentations of TB in immunocompromised patients, particularly those receiving immunosuppressive medications, and the potential overlap in clinical manifestations between TB and BD, especially in extrapulmonary TB or systemic involvement, we have chosen to present this case. Considering the limited literature on TB in BD patients, increasing awareness among healthcare providers about this condition can aid in early diagnosis and better management of this patient population.

## 2 Case History

A 43‐year‐old man, with a history of substance abuse (oral opium), was admitted to the hospital 16 years ago, presenting with symptoms of severe weight loss from 88.5 to 49.5 kg, abdominal pain, and shortness of breath. He had been diagnosed with BD at the age of 13, with ocular involvement along with oral and genital ulcers. He was undergoing treatment with a daily dose of prednisone. The patient reported a prior history of treatment with azathioprine and cyclophosphamide for BD. Prior to initiation of pharmacotherapy for BD, the patient underwent screening for TB, which had a negative result. Abdominal examination revealed distension, palpable tenderness, decreased bowel sounds on auscultation, and signs of peritonitis. Crackles and wheezing were detected on chest auscultation, particularly in the upper lung regions.

### 2.1 Diagnostic Findings

Following consultations with gastroenterology and infectious disease specialists, the patient underwent abdominal and chest imaging. Abdominal X‐rays indicated intestinal obstruction and adhesions. Initial differential diagnoses included progressive BD, vasculitis, and possibly Crohn's disease. Subsequently, the patient underwent bowel transit and abdominopelvic computed tomography (CT) scans, revealing a small bowel stricture. Additionally, chest X‐rays showed a cavity and calcification. Further evaluation included a sputum culture identifying acid‐fast bacilli, and a chest CT scan, which revealed scattered fibrotic bands on the apices of both lungs, mainly on the left side, as well as pleural thickening and calcification associated with inactive pulmonary TB.

### 2.2 Treatment and Follow‐Up

The patient was diagnosed with pulmonary TB and initiated on a multi‐drug regimen consisting of isoniazid, ethambutol, rifampicin, and pyrazinamide for a duration of 6 months, resulting in significant improvement of symptoms.

One year after the completion of the treatment, the patient was readmitted with symptoms of headache, fever, severe chills, and convulsions. Neurological examinations revealed hemiparesis, prompting a brain magnetic resonance imaging (MRI), which confirmed the presence of a brain abscess. Consequently, the patient underwent surgery to remove the brain abscess. Following the surgery, the patient received a 1‐year course of TB treatment regimen, during which the patient demonstrated satisfactory adherence to the treatment and reported no specific adverse effects. After completion of TB treatment, the patient was regularly followed up for 2 years, during which no complications arose, and his symptoms resolved completely.

**FIGURE 1:**
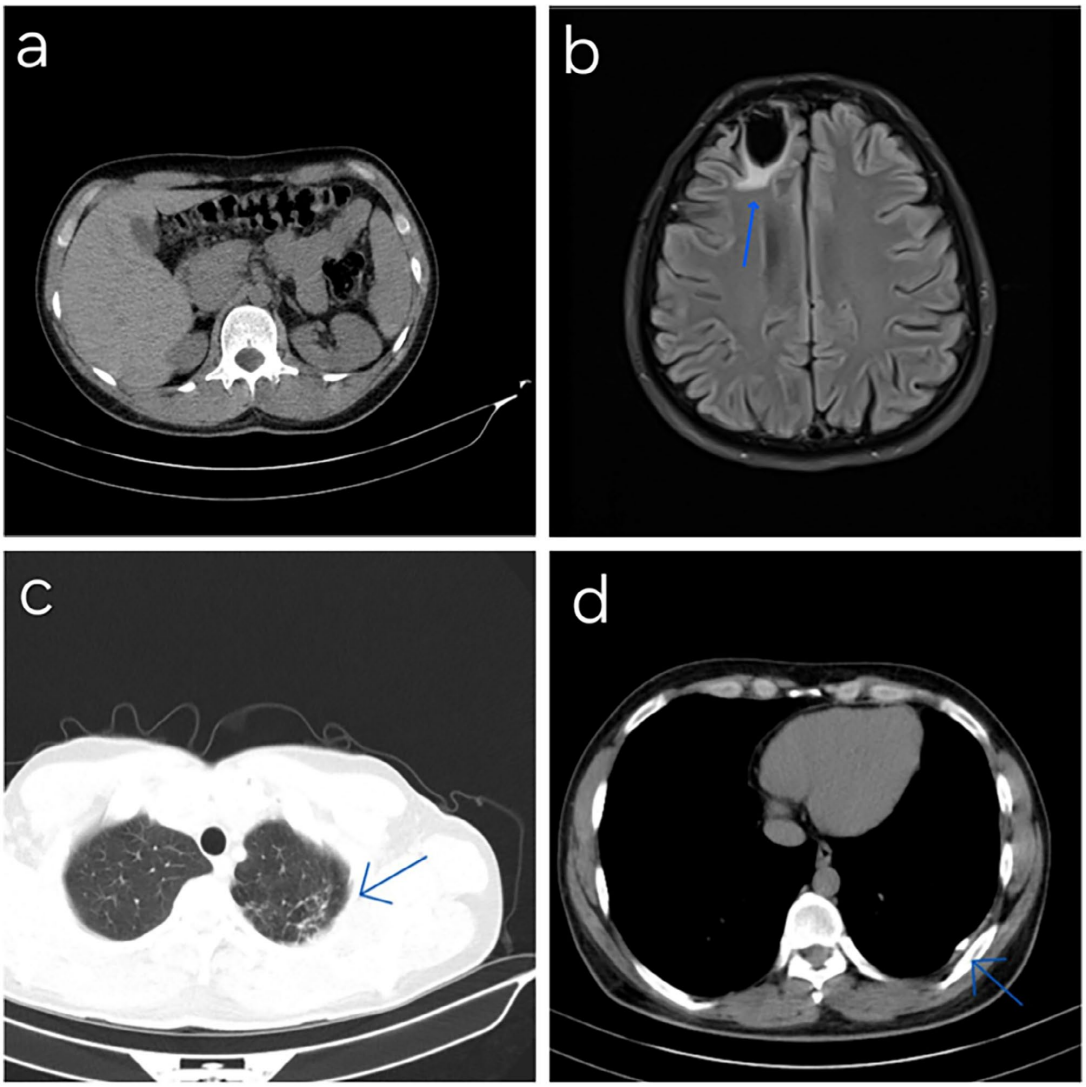
(a) Abdominopelvic CT scan revealing normal solid organ appearance, absence of focal lesions, kidney or gallbladder stones, with no signs of bowel wall thickening or obstruction. There is no presence of lymphadenopathy or abscess lesions. (b) Brain MRI findings reveal a focal region of approximately 19.25.11 mm with edema and encephalomalacia in the right frontal lobe (blue arrow), as sequelae from the patient's previous history of abscess and surgical drainage. No mass lesions or midline shifts were observed, with an intact ventricular system and patent basal cisterns. Normal cerebellum, brainstem, and intracranial vessels were noted. (c, d) Comprehensive assessment on high‐resolution CT scan revealing fibrotic bands in lung apices, scattered mainly on the left side, with pleural thickening and occasional calcifications on the posterior pleural surfaces (blue arrows). Normal lung volume and density, patent airways, and unremarkable mediastinal structures are, suggestive of an old and inactive tuberculosis infection.

**FIGURE 2:**
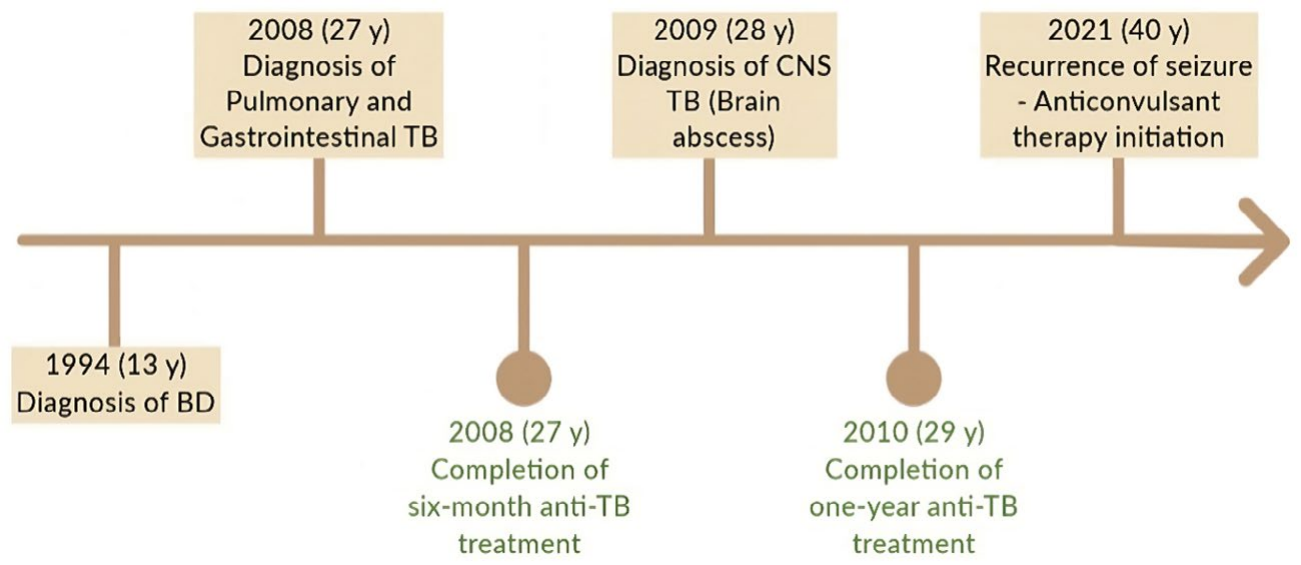
Timeline of case presentation description.

In 2021, the patient experienced a further seizure, prompting the performance of brain MRI, abdominopelvic CT scan, and chest CT scan, all of which revealed no acute pathological findings (Figure 1). Subsequently, sodium valproate was prescribed for the patient. His medical history, including previous brain abscess surgery and a brain lesion, influenced this decision. In addition, the patient had mood disorders, and the mood‐stabilizing effects of sodium valproate were beneficial in this case (Figure 2).

A recent favorable 16‐year follow‐up evaluation of the patient demonstrated a significantly satisfactory outcome, indicating that the patient's quality of life has improved. Currently, the patient's BD symptoms are under control. Abdominal radiography revealed no detectable abnormalities. Additionally, the chest X‐ray showed the absence of any pulmonary complications, with only a few fibrotic lesions observed in the areas where previous lesions had existed. The MRI indicated the presence of some brain lesions. The patient was referred to a psychiatrist for mood disorders and is currently receiving treatment.

## 3 Discussion

This article presents a case of miliary TB with multiple organ involvement, including lungs, intestine, peritoneum, and brain, in an adult male with BD. The simultaneous occurrence of TB in three organs is a rare phenomenon and, to the best of our knowledge, no studies have reported a patient with BD and concurrent TB involvement in the lungs, GI, and CNS organs. Althoug, this patient had multiorgan involvement, the lungs did not exhibit a miliary shadow pattern and only showed involvement in the apexes. The presence of BD along with the use of immunosuppressive drugs further complicated the early diagnosis. Early identification of this condition depends on a detailed medical history and a high level of clinical suspicion. Given that BD can present symptoms similar to those of TB, this poses a considerable diagnostic challenge. Studies have documented cases of TB affecting the CNS and GI organs in BD patients, underscoring the difficulty in diagnosing TB in this population. These findings are outlined in Table 1. Additionally, a condition known as pseudo‐Behçet, which can mimic BD, has been described in the literature. This condition is attributed to hypersensitivity reactions to *M. tuberculosis* bacteria and is characterized by symptoms such as reactive arthritis, erythema nodosum, and orogenital ulcers [7]. Among diagnostic tests, T‐SPOT.TB has demonstrated greater accuracy compared to the tuberculin skin test [3]. This comprehensive literature review provides an overview and detailed examination of studies pertaining to TB in patients with BD, emphasizing the significance and unanticipated complexities associated with initial misdiagnosis (Table 1).

**TABLE 1:** A review of studies on adults with BD who developed TB during treatment and patient characteristics.

Study	Number of subjects	Age	Gender	Symptoms	Radiological findings	Diagnosis	Management
Efthimiou et al. (1988) [40]	3	37	Female	Cough, right‐sided pleuritic chest pain, recurrent small hemoptyses and weight loss	Patchy shadowing with cavitation in both upper lobes	Pulmonary tuberculosis	Rifampicin, isoniazid, ethambutol, and pyrazinamide
		28	Female	Fever, malaise, cough and left‐sided pleuritic chest pain	Left‐sided pleural effusion		Rifampicin, isoniazid, and ethambutol
		31	Female	Fever, night sweats, and right upper chest pain	Patchy shadowing and cavitation in the right upper lobe		Rifampicin, isoniazid, and ethambutol
Kapan et al. (2006) [44]	1	60	Male	Multiple ileal perforations	Multiple nodular opacity on both lungs	Miliary tuberculosis	Isoniazid, rifampicin, ethambutol, and pyrazinamide
Iliopoulos et al. (2006) [33]	1	21	Male	Left‐sided hemiparesis, vertigo, and severe headache	High signal intensity lesions involving bilateral basal ganglia, head of right caudate nucleus, and adjacent white matter tracts of the right frontal lobe	Tuberculous meningoencephalitis	Isoniazid, rifampicin, ethambutol, and pyrazinamide
Skvara et al. (2009) [10]	1	24	Female	Fever, shivering, vigorous coughing, and night sweats	Bi‐hilar lymphadenopathy and disseminated nodular densities	Primary miliary tuberculosis	Isoniazid, rifampicin, ethambutol, and moxifloxacin
Gönen et al. (2012) [43]	1	22	Male	Fever, headache, and fatigue	Miliary lung tuberculosis Tuberculous granuloma and tuberculous meningitis Widespread lytic lesions in the vertebra corpuses and the tissue swelling adjacent to the vertebra were consistent with Pott's disease and spinal granuloma	Disseminated tuberculosis (testicular tuberculosis, miliary tuberculosis, tuberculosis meningitis, spinal granuloma, and Pott's disease)	Isoniazid, streptomycin, pyrazinamide, and rifampicin
Eun et al. (2016) [34]	1	14	Female	Headache, remittent fever, vomiting, and intermittent cough	Diffuse micronodular pattern of miliary pulmonary tuberculosis Diffuse pial enhancement in the brainstem, left precuneus, and right opercular gyrus, and multifocal enhancing lesions predominantly within the bilateral centrum semiovale and right parietotemporal white matter in the T2‐weighted images	Neuro‐Behcet's syndrome and miliary pulmonary tuberculosis	Isoniazid, ethambutol, pyrazinamide, rifampicin, and dexamethasone
Shen et al. (2019) [19]	1	44	Female	Fever	Thickening of the ileocecal wall	Ulcerative intestinal TB	Isoniazid, rifapentine, ethambutol, and pyrazinamide
Sekkat et al. (2020) [41]	1	47	Female	Fever, weakness of the 2 uppers limbs	Several micronodules, uniformly distributed throughout the lung (hematogenous distribution), related to a tuberculous miliary on chest CT scan Multiple punctiform and nodular supra and infratentorial parenchymal lesions, predominant at the gray‐white matter junction, with mild hypersignal on T2 WI and FLAIR Brain MRI, enhanced after contrast	Cerebral and pulmonary tuberculous miliary	Isoniazid, rifampicin, pyrazinamide, and ethambutol
Toriu et al. (2023) [11]	1	48	Male	General malaise	Granular shadows in bilateral lungs	Miliary TB	Isoniazid, rifampicin, ethambutol, and pyrazinamide

While TB typically affects the lungs, it can also manifest anywhere else in the body, leading to extrapulmonary TB [8]. Miliary TB encompasses both pulmonary and extrapulmonary manifestations and spreads through hematogenous dissemination. It is particularly prevalent in immunocompromised individuals and can lead to severe and fatal consequences [9, 10, 11].

Extrapulmonary TB accounts for a significant proportion of TB cases worldwide. Based on the 2022 ECDC Report, a total of 33,148 TB cases were reported in 2020. Of those cases, 21.5% were classified as extrapulmonary TB, 73.1% as pulmonary TB, and the remaining cases were either concurrent pulmonary‐extrapulmonary TB or lacked a reported TB site. Various risk factors have been identified for the development of extrapulmonary TB, including age (under 15 and over 65 years old), female sex, migration from high‐incidence TB countries, and immunosuppression. However, there is limited information on other epidemiological, clinical, or microbiological factors contributing to extrapulmonary TB development [12, 13].

GI TB is a specific form of extrapulmonary TB, accounting for approximately 11%–16% of all extrapulmonary TB cases and 1%–3% of all TB cases [14, 15, 16]. Furthermore, 6%–38% of patients with intra‐abdominal TB may also have pulmonary TB [17, 18]. In both developing and developed countries, GI TB has been associated with several factors, including treatment with anti‐tumor necrosis factor‐alpha (anti‐TNFα) agents, solid‐organ transplantation, particularly cardiac, liver, renal, and renal–pancreas transplants [19, 20, 21, 22, 23]. Notably, there have been reports of GI TB in the immunocompetent population, raising concerns about its prevalence in this group [24].

The diagnosis of GI TB presents a complex challenge due to its non‐specific manifestations, including fever, significant weight loss, anorexia, and night sweats [19, 25]. This often leads to delayed treatment and a poor prognosis. GI TB can be acquired through various mechanisms, such as hematogenous or lymphatic spread, ingestion of bacteria from sputum or contaminated milk/food, and direct spread from adjacent organs [26, 27, 28]. In the present case, hematogenous spread likely accounts for the dissemination of the disease to the digestive system.

Manifestations of GI TB can take different forms, including ulcerative, ulcero‐hypertrophic, and hypertrophic. It can also mimic other GI disorders, including Crohn's disease, peptic ulcers, and malignancies [19, 29, 30]. Symptoms such as abdominal pain, fever, and fatigue are common in GI TB and Crohn's disease. However, mild fever, weight loss, and night sweats are typical of GI TB, whereas Crohn's disease is characterized by malabsorption and protein loss. The imaging patterns of these conditions differ on CT scans, with GI TB showing focal involvement and Crohn's disease exhibiting segmental involvement [31]. Given the concomitant lung involvement on radiographic images, positive sputum culture, the patient's use of immunosuppressive drugs, the high prevalence of TB in Iran, and focal small intestine involvement on abdominopelvic CT scan, the patient was diagnosed with GI TB. The patient's symptoms also improved following a TB treatment regimen.

TB of the CNS is prevalent in developing countries, particularly among individuals with HIV [32]. CNS TB can manifest as tuberculosis meningitis, tuberculoma/brain abscess, spinal cord involvement, or a combination of these conditions [33, 34, 35]. CNS TB commonly results from hematogenous dissemination, leading to the formation of small caseous tubercles within the parenchyma and meninges, known as Rich foci. This process can lead to localized inflammatory reactions or the spread of infection throughout the nervous system. Rupture of these Rich foci can cause the release of bacilli from the leptomeninges, thus triggering widespread inflammation. Moreover, caseous necrosis within Rich foci may lead to their enlargement and the generation of inflammatory exudate in the surrounding region [35, 36]. The development of a brain abscess is a rare manifestation, typically appearing as a single cavity, but it can also occur as multiple cavities [37]. It can form in cortical–subcortical areas, the cerebellum, and the central gray nuclei of the brain, likely due to hematogenous spread. Abscess formation may manifest with symptoms including headache, convulsion, nausea, and decreased level of consciousness [38]. In this regard, Iliopoulos et al. reported a known case of BD characterized by fever, left‐sided hemiparesis, vertigo, and severe headache. Brain MRI revealed a lesion in the right frontal lobe. The patient showed symptom improvement within 1 week of initiating a TB treatment regimen [33]. Our patient presented with symptoms such as headache and convulsion, which is, to the best of our knowledge, the first case of BD with CNS TB reported in the literature.

Treatment of brain abscesses involves a combination of medical and surgical interventions, with the surgical approach involving excision through craniotomy or aspiration [38]. In this patient, the craniotomy method was used, resulting in complete removal of the abscess.

There is a reciprocal relationship between TB and BD. Patients with BD are more prone to developing active TB and experiencing involvement at multiple sites. Consequently, TB patients with concurrent BD may present with systemic symptoms, such as night sweats, fever, and unexplained weight loss, as well as infection site‐specific manifestations [34, 39, 40]. *M. tuberculosis* can trigger the development of BD, while BD, in turn, heightens susceptibility to TB by compromising cellular immunity. Furthermore, individuals with BD and LTBI may progress to active TB following the administration of glucocorticoids and immunosuppressive or biologic agents. Anti‐TNFα agents may increase the risk of reactivation or progression of active TB. As a result, it is recommended to screen and treat LTBI before beginning treatment with this group of drugs. However, there is no data on LTBI management in BD patients [3, 10, 11, 34, 40, 41].

Standard treatment for TB involves drug therapy. For pulmonary TB, a four‐drug regimen comprising isoniazid, rifampicin, ethambutol, and pyrazinamide is typically administered for 2 months, followed by a two‐drug regimen of rifampicin and isoniazid for an additional 4 months. Extrapulmonary and disseminated TB patients, such as the case described, usually require longer treatment durations. TB affecting the CNS necessitates 12 months of drug treatment. Furthermore, treatment duration and approach for disseminated TB should be tailored based on the patient's clinical progress [42].

As previously indicated, the use of immunosuppressive and anti‐TNFα drugs in various diseases can lead to the activation of LTBI. Hence, it is advisable to screen patients for the presence of *M. tuberculosis* before initiating immunosuppressive therapy and to provide treatment if the infection is detected [10, 41, 43]. Prophylactic treatment is also recommended for patients undergoing organ transplantation [10, 41].

## 4 Conclusion

In conclusion, this case highlights the importance of considering pulmonary and extrapulmonary TB in patients with complex medical conditions, especially those with autoimmune diseases like BD. Early diagnosis and treatment are crucial for improving patient outcomes and reducing the risk of serious complications such as brain abscesses. Clinicians should implement screening protocols for LTBI in BD patients and closely monitor them for any signs of TB infection. Furthermore, there is a need for future research to explore safer immunosuppressive therapies and develop better diagnostic tools for early detection of TB in these high‐risk patient populations. Finally, this case report aims to raise awareness among health care providers and improve patient management for individuals with similar medical conditions.

## Author Contributions

**Homina Saffar:** data curation, writing – original draft. **Pooria Sobhanian:** data curation, writing – original draft. **Shahriar Alian:** conceptualization, supervision. **David Darvishnia:** conceptualization. **Mansoureh Baradaran:** writing – review and editing.

## Ethics Statement

The authors have nothing to report.

## Consent

The written informed consent for publication was obtained from the patient.

## Conflicts of Interest

The authors declare no conflicts of interest.

## Data Availability

The data in this article will be shared on request.
